# Interleukin-8 for Diagnosis of Neonatal Sepsis: A Meta-Analysis

**DOI:** 10.1371/journal.pone.0127170

**Published:** 2015-05-21

**Authors:** Min Zhou, Shupeng Cheng, Jialin Yu, Qi Lu

**Affiliations:** Department of Neonatology, Children’s Hospital of Chongqing Medical University, Ministry of Education Key Laboratory of Child Development and Disorders, Chongqing, China; univeristy of alabama at birmingham, UNITED STATES

## Abstract

**Background:**

Neonatal sepsis (NS) is a life-threatening disorder and an important cause of morbidity and mortality in neonates. Previous studies showed that interleukin 8 (IL-8) may effectively and rapidly diagnose NS.

**Objective:**

We conducted the systematic review and meta-analysis to investigate the diagnostic value of the IL-8 in NS.

**Methods:**

The literature was searched in PUBMED, EMBASE, Cochrane Library, CNKI, VIP and other Chinese Medical Databases during October 1998 to January 2014 using set search criteria. Each included study was evaluated by quality assessment of diagnostic accuracy studies tool. Two investigators independently extracted the data and study characteristics, and disagreements, if any, were resolved by consensus. Meta-disc software was used to calculate the pooled sensitivity, specificity and summary diagnostic odds ratio (SDOR), I² or Cochrane Q to test heterogeneity, and meta-regression to investigate the source of heterogeneity. Funnel plots were used to test the potential presence of publication bias. False-positive report probability (FPRP) was calculated to confirm the significance of the results.

**Results:**

Eight studies (548 neonates) were included in this meta-analysis. The pooled sensitivity and specificity of IL-8 were 0.78 and 0.84, respectively, which had moderate accuracy in the diagnosis of NS. The pooled diagnostic odds ratio (DOR) and area under curve (AUC) was 21.64 and 0.8908 (Q*=0.8215), respectively. The diagnostic threshold analysis showed that there was no threshold effect. The meta-regression analysis showed the cut-off, QUADAS and onset time have no effect on the heterogeneity. The funnel plots showed the existence of publication bias.

**Conclusion:**

Meta-analysis showed IL-8 had a moderate accuracy (AUC=0.8908) for the diagnosis of NS. IL-8 is a helpful biomarker for early diagnosis of NS. However, we should combine the results with clinical symptoms and signs, laboratory and microbial results.

## Introduction

Neonatal sepsis (NS) is a life-threatening disorder and an important cause of morbidity (1 to 10 per 1000 live births) and mortality (15% to 50%) in neonates, especially in preterm [1, 2].

NS can be defined both clinically and/or microbiologically as a systemic inflammatory response caused by infection. The gold standard of diagnosis NS is the positive culture of blood and/or sterile body fluid, but it is less sensitive and takes approximately 48 to 72 hour [3, 4]. Early clinical signs of NS such as fever, tachycardia, and drowsiness are nonspecific, and it is easy to misdiagnose with other common diseases such as pneumonia, respiratory distress syndrome, and intracranial hemorrhages [5].

Laboratory tests such as white blood cell count, neutrophil percentage, platelet count, and C-reactive protein (CRP) are non-specific in NS. During the first hours of NS, reliable biomarkers of infection are absent. Therefore, to avoid the adverse outcomes such as septic shock, multiple organ dysfunction syndrome (MODS) and even death, pediatricians often treat with a broad-spectrum antibiotic and prolong treatment with empirical antibiotics, exposing many neonates to unnecessary anti-infectious treatments [2, 6, 7]. Therefore, urgent needs for reliable diagnostic biomarkers for early diagnose neonatal sepsis [8].

Humoral and cellular systems are activated in the first hours of neonatal sepsis, various molecules such as Interleukin-6 (IL-6), Procalcitonin (PCT), C-reactive protein (CRP), and IL-8 released in the serum which mediated the host response to bacterial infection. Interleukin-8 (IL-8) is a pro-inflammatory cytokine and is predominantly produced by monocytes, macrophages, and endothelial cells. IL-8 regulates the migration and activation of leukocytes, whose level evaluate promptly within 1–3 hours of infection and its half-life is less than 4 hours [9–11]. Many studies have showed IL-8 is an early-phase biomarker for diagnosis of NS, and IL-8 test may be a valid non-invasive, effective, and rapid method for diagnosis NS [8–11]. Several potential biomarkers such as Interleukin-6 (IL-6), Procalcitonin (PCT) and C-reactive protein (CRP) have been investigated their validity for early diagnose NS. However, there are no large sample multi-center studies, no systematic review and meta-analysis on interleukin-8 for diagnosis of neonatal sepsis, therefore, we conducted this systematic review and meta-analysis to assess the validity of IL-8 test for early diagnosis neonatal sepsis, and systematically and quantitatively evaluate all published studies about the diagnostic value of IL-8 test for NS.

## Methods

### Literature Search and Selection of Studies

Computer-aided literature search was carried out in PUBMED, EMBASE, Cochrane Library, CNKI, VIP, and other Chinese Medical Database for relevant citations published during October 1998 to January 2014, and without language restrictions. The search terms were “neonate”, “sepsis”, “septicemia”, “biomarker” and “interleukin-8”. Both the authors were examined the references of the fully retrieved articles.

The inclusion criteria were: (1) studies which assessed the diagnostic accuracy of the IL-8 test on NS; (2) studies included case group: culture or clinical sepsis; control group: neonates have systemic inflammatory response or healthy neonates [12]; (3) studies provided both sensitivity and specificity or sufficient information to construct 2×2 tables; (4) studies containing only neonates (from birth to 28 days); and (5) articles which evaluated IL-8 levels were included in this study. The gold standard for the diagnosis of NS involves microbial culture of blood or other sterile body fluids. Furthermore, the change of the IL-8 in the research sample is an index test for the diagnosis of NS. The exclusion criteria were: Studies which included only healthy neonates, neonates without probable infection, non-serologic biomarkers. We also excluded animal experiments, reviews, correspondences, case reports, expert opinions, and editorials. These articles were reviewed by two investigators independently, and disagreements, if any, were resolved by consensus.

### Data Extraction

The information was extracted from the selected articles included first author, year of publication, study method, region, measure method of IL-8, diagnostic cut-off point and time, sample size, sensitivity, specificity, and so on. The optimal cut-off point obtained with the ROC method of each included study. Accurate true-positive, false-positive, false-negative, and true-negative results were extracted to construct 2×2 table at a specific time for each study. If any additional information needed was not reported in the published articles; hence, an electronic-mail was sent to the corresponding authors asking further information. If there was no reply, such articles were excluded from the meta-analysis.

### Assessment

The methodological quality of the studies was assessed using guidelines published by the quality assessment of diagnostic accuracy studies (QUADAS) tool including 11 questions. Questions with “yes”, “no”, and “unknown” answer, were scored as 1, -1, and 0, respectively [13].

### Statistical Analysis

Meta-Disc 1.4 software was used for statistical analysis and RevMan 5.0 was used to analysis the publication bias (funnel plots) [14]. Sensitivity, specificity, positive likelihood ratio (PLR), and negative likelihood ratio (NLR) with corresponding 95% confidence intervals (CI) were calculated for each study. We used the Random Effects Model to calculate the pooled sensitivity, specificity, and DOR [15–17]. Heterogeneity among included studies was assessed using the Cochrane Q statistics and I^2^ test [17]. *I* ^2^ can be readily calculated from basic results obtained from a typical meta-analysis as *I* ^2^ = 100%×(*Q*—df)/*Q*, where *Q* is Cochran's heterogeneity statistic and df the degrees of freedom. Normally, I^2^ lies between 0% and 100%. If I^2^<50%, then there is more homogeneity among studies during meta-analysis; whereas I^2^>50%, then there is more heterogeneity among studies. A value of 0% indicates no observed heterogeneity and larger values show increased heterogeneity [16, 17]. Hence, we examined characteristics of included studies. These results were summarized to construct a summary receiver operator characteristic (SROC) curve, which showed the relationship between sensitivity and specificity (proportion of false positives). Q* value, was defined where the SROC curve crosses the anti-diagonal from (0; 1) to (1; 0) of the SROC space; hence TPR = 1 − FPR at Q*, and so the probability of an incorrect result from the test is the same for cases and non-cases [18]. Meanwhile, the area under SROC curve was also calculated to show the diagnostic accuracy of IL-8 test [18–21]. As the cut-off range from 0.65 to 300 (pg/ml), we did excluded the data of which cut-off equal 0.65 (pg/ml) to do sensitivity analysis.

To avoid false positive findings, the false-positive report probability (FPRP) values and statistical powers were performed using the method reported by Wacholder et al [22, 23]. Firstly we set 0.2 as an FPRP threshold and selected 6, 15 and 22 as most likely DOR. Secondly FPRP analysis with prior probabilities of 0.25, 0.1, 0.01, 0.001 and 0.0001 were obtained, and when FPRP value less than 0.2 were considered as noteworthy associations. The Excel spreadsheet provided by Wacholder et al. was used to calculate statistical power and FPRP values (http://jnci.oxfordjournals.org/content/96/6/434/suppl/DC1).

## Results

### Characteristic and Quality of the Included Studies

**Fig 1:**
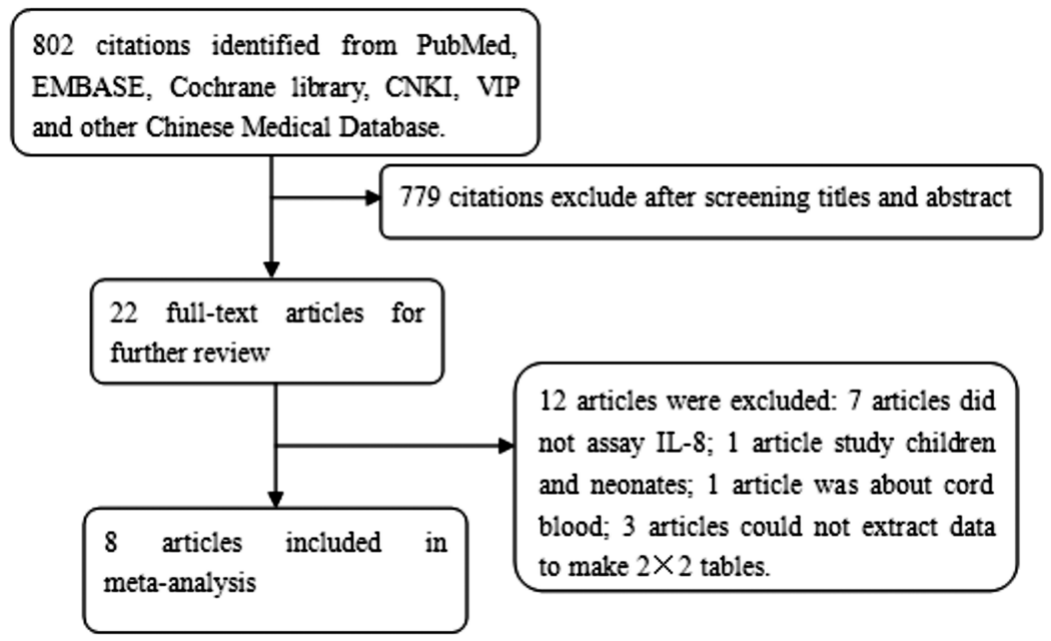
Flow chart of the process of the articles identified and included.

**Fig 2:**
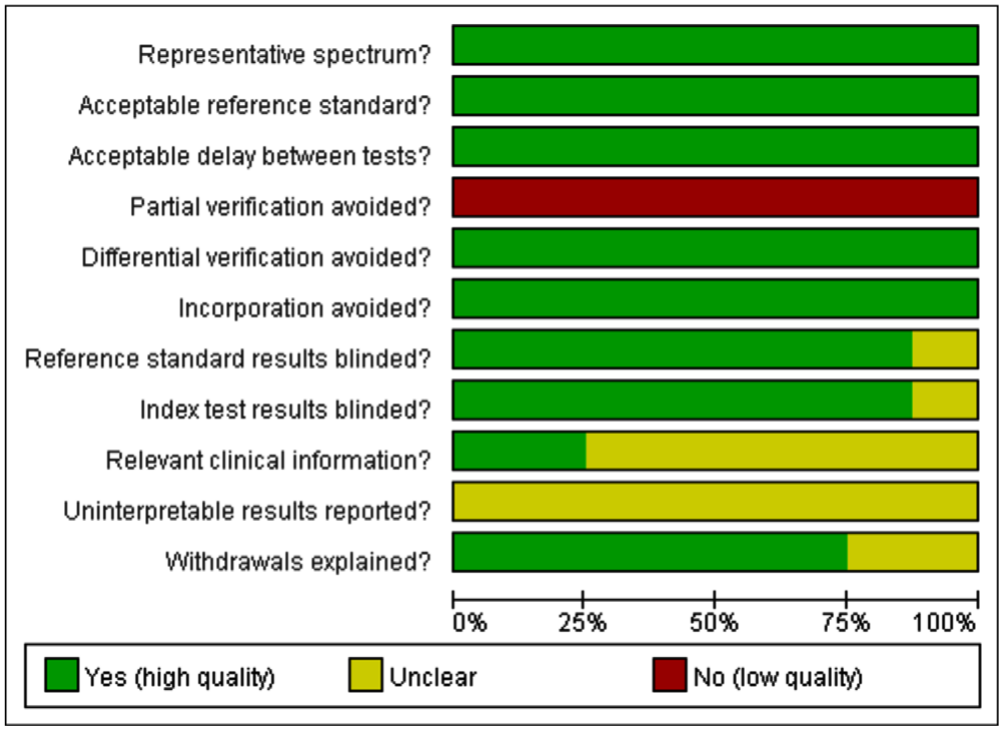
QUADAS results about the level of risk of bias for each included study.

Twenty two potential articles regarding IL-8's role in diagnosing NS were identified. Only eight articles met the inclusion criteria. Fig 1 shows the selection process of studies. The detailed characteristics of the included studies are presented in Table 1. The true positive, false positive, false negative, true negative, sensitivity, and specificity of each article were shown in Table 2. The QUADAS result about the level of risk of bias for each included study was shown in Fig 2.

**Table 1:** Main characteristics of studies included in the meta-analysis.

Studies	Study population	Region	N	Measure method	Cut-off (pg/ml)	Tine	Sepsis diagnosis
Prashant^1^ (2013)	Case: neonates with sepsis	India	100	ELISA	70.86	EONS:41	Culture; clinical
	Control: neonates without sepsis					LONS:9	
Kocabaş^2^ (2007)	Case: neonates with sepsis	Turkey	55	ELISA	0.65	EONS:13	Culture; clinical
	Control: neonates without sepsis					LONS:13	
Laborada^3^ (2003)	Case: neonates with sepsis	USA	105	CLIAA	100	EONS:20	Culture; clinical
	Control: neonates without sepsis					LONS:28	
Santana^4^(2003)	Case: neonates with sepsis	Spain	40	CLEIA	63	EONS:12	Culture; clinical
	Control: neonates without sepsis					LONS:8	
Martin^5^ (2001)	Case: neonates with sepsis	Sweden	32	CLIA	70	EONS:<48hour	Culture; clinical
	Control: neonates without sepsis						
Nupponen^6^ (2001)	Case: neonates with sepsis	Finland	35	ELISA	50	LONS	Culture; clinical
	Control: neonates without sepsis						
Boskabad^7^ (2010)	Case: neonates with sepsis	Iran	80	ELISA	60	EONS	Culture; clinical
	Control: neonates without sepsis						
Berner^8^ (1998)	Case: neonates with sepsis	Germany	101	Double sandwich EIA	300	EONS	Culture; clinical
	Control: neonates without sepsis						

**Table 2:** Tp, Fp, Fn, Tn, Se, Sp, time, and QUADAS of included studies for the diagnosis of NS. (3 CLIAA (cytoscreen immunoassay kits) 5 CLIA (Immulite; Diagnostic Products Corporation, Los Angeles, CA) 6 ELISA (Quantikine, R&D Systems, Minneapolis, MN)).

Studies	Tp	Fp	Fn	Tn	Se	Sp	QUADAS score
Prashant	39	15	11	35	0.780	0.700	8
Kocabaş	9	4	17	25	0.346	0.862	4
Laborada	36	19	12	38	0.750	0.667	7
Santana Reyes	12	1	8	19	0.600	0.950	7
Martin H	11	6	1	14	0.917	0.700	7
Nupponen	20	0	2	13	0.909	1.000	7
Boskabadi	36	0	2	42	0.948	1.000	8
Berner	32	5	3	61	0.910	0.930	6

True positive (Tp), False positive (Fp), False negative (Fn), True negative (Tn), Sensitivity (Se), and Specificity (Sp). QUADAS is a system to evaluate the articles included in this meta-analysis.

### Accuracy of the IL-8 Test on NS

**Fig 3:**
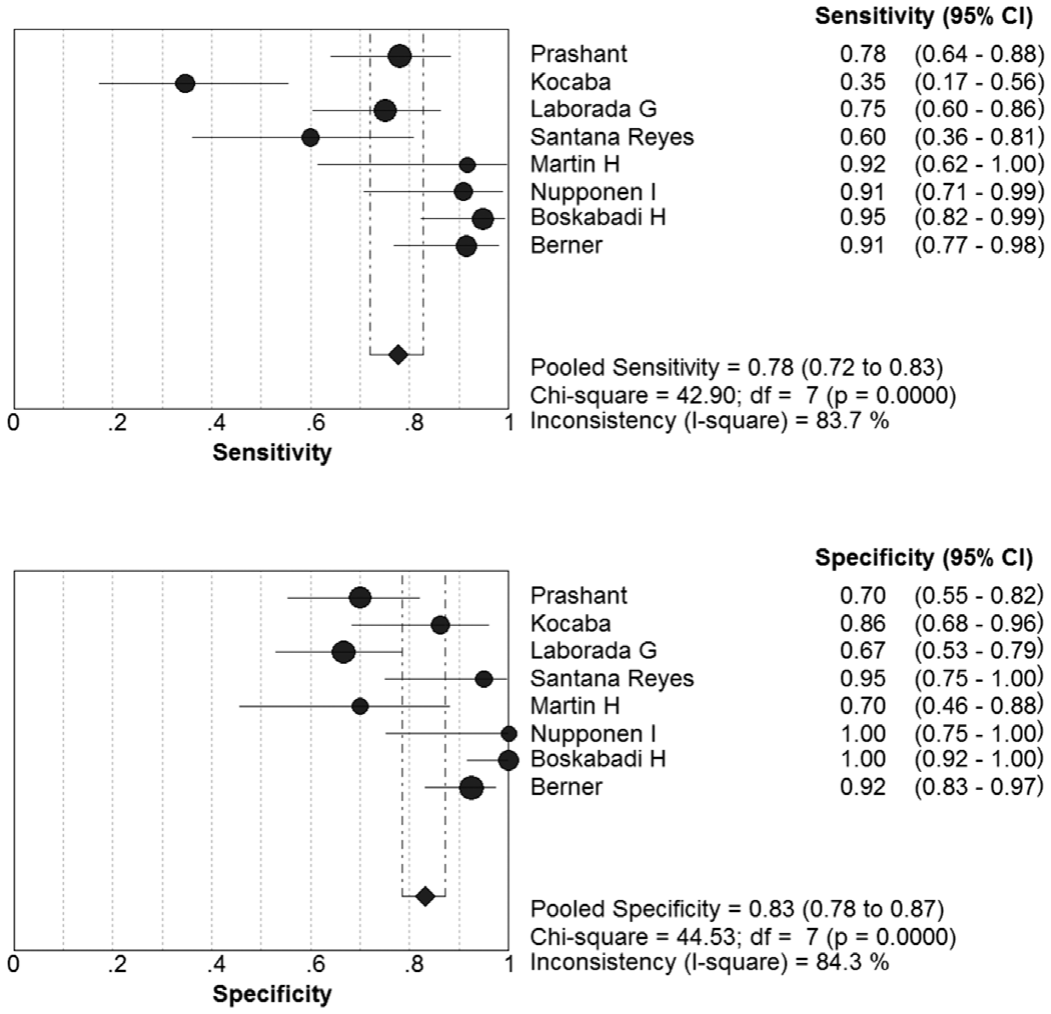
Forest plots of the pooled sensitivity and specificity of the IL-8 to diagnose NS.

Eight articles met the inclusion criteria. The sensitivity ranged from 0.35 to 0.95 (pooled sensitivity: 0.78, 95% CI: 0.72 to 0.83); whereas specificity ranged from 0.67 to 1.00 (pooled specificity: 0.84, 95% CI: 0.79 to 0.88) (Fig 3). The present study found significant heterogeneity among studies (sensitivity, I^2^ = 83.7%; specificity, I^2^ = 85.1%), which indicated that might the study population; measure methods and other covariates were responsible for it.

**Fig 4:**
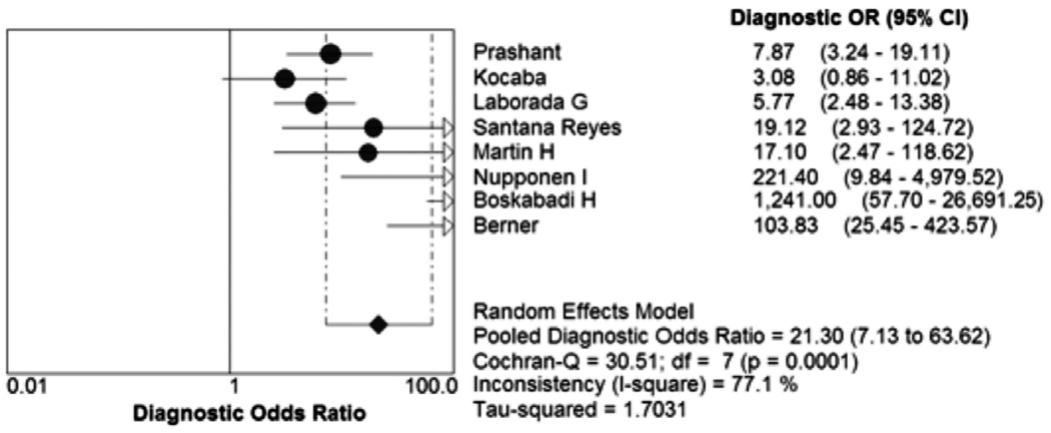
Forest plots of diagnostic odds ratio (DOR) of the IL-8 to diagnose NS.

**Fig 5:**
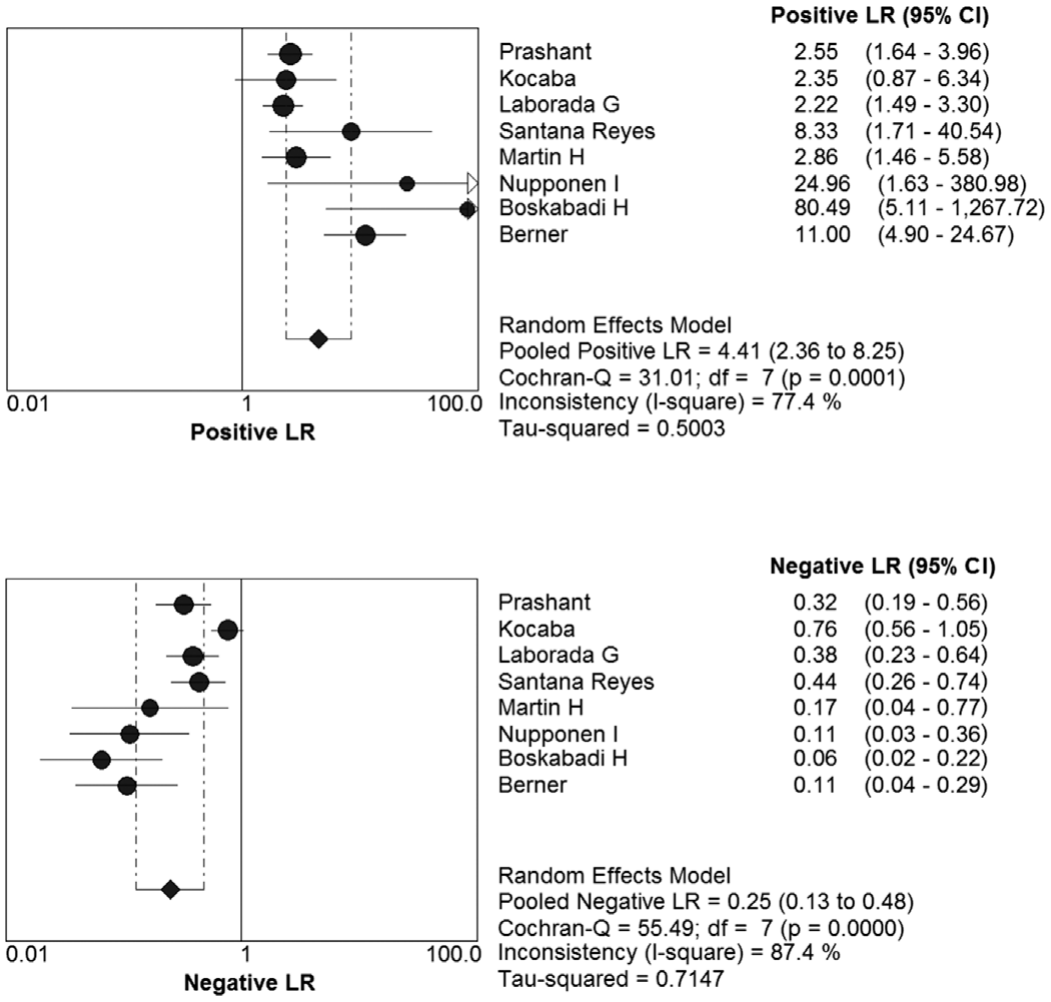
The summary receiver operating characteristic (SROC) curve for assessment of the IL-8 to diagnose NS.

**Fig 6:**
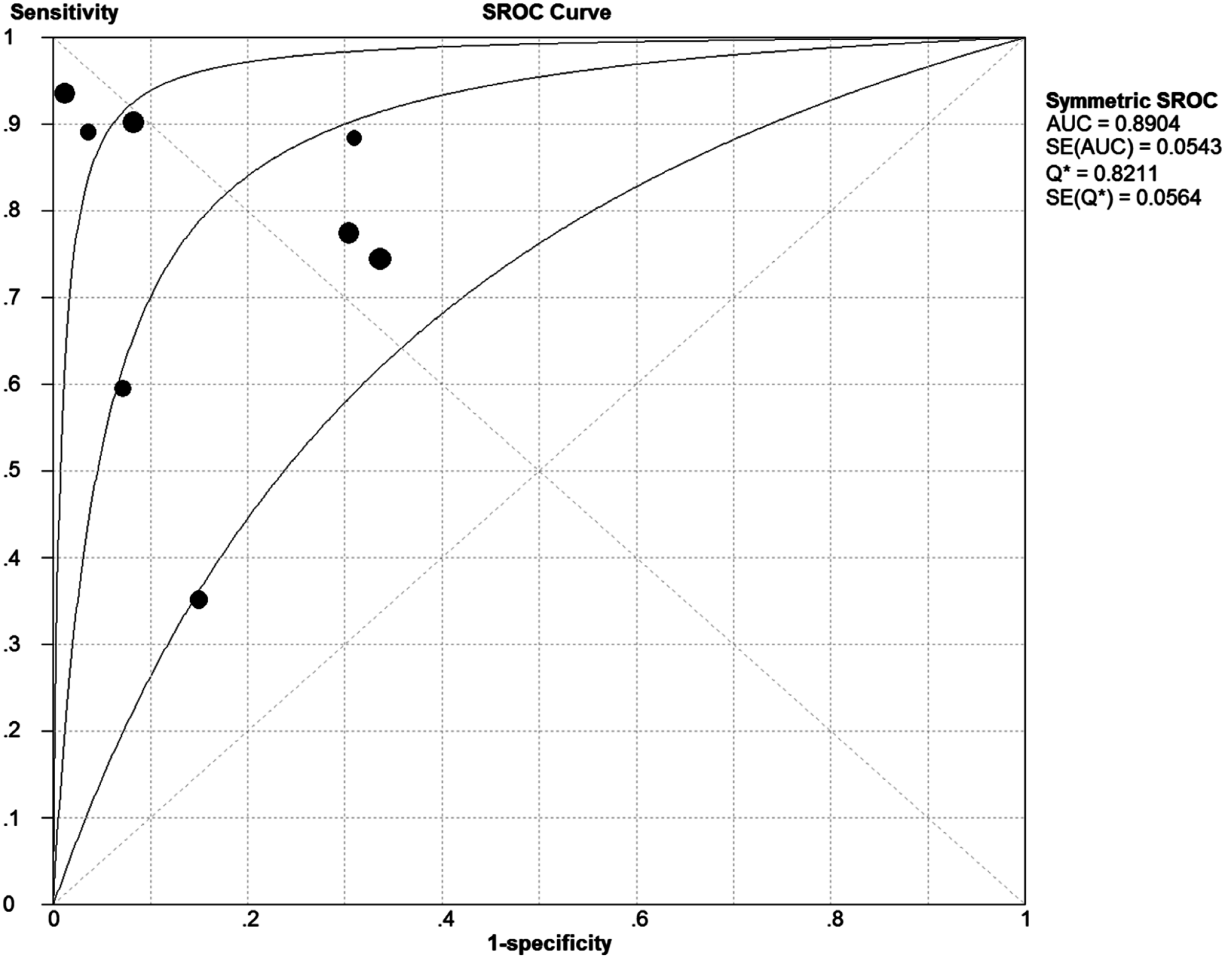
The positive LR and negative LR of IL-8 test on diagnosis NS.

The pooled Diagnostic Odds Ratio of IL-8 was 21.64 (95% CI: 7.37 to 63.54) (Fig 4). Among all these studies, a significant heterogeneity (I^2^ = 77.3%) was detected. The corresponding SROC curve was plotted in Fig 5, which showed the AUC was 0.8908 with standard error of 0.0539 and the Q* value was 0.8215 with standard error of 0.0560. It also showed a moderate accuracy of IL-8 test to diagnose NS. The pooled PLR value of IL-8 test was 4.58 (95% CI: 2.44 to 8.60); whereas, pooled NLR value of IL-8 test was 0.25(95% CI: 0.13 to 0.48) (Fig 6).

**Fig 7:**
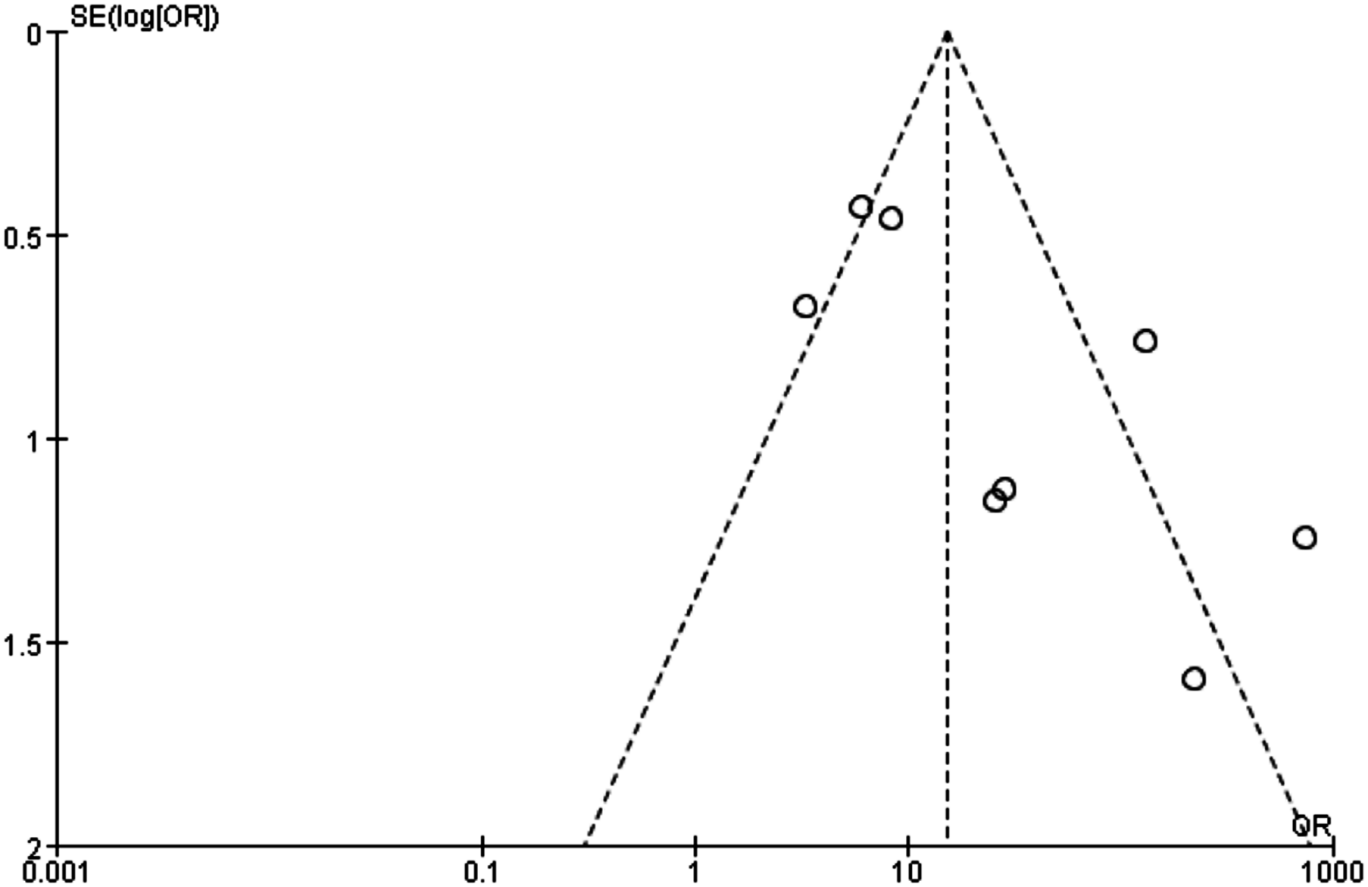
Funnel plot to estimate the publication bias of the meta-analysis.

The funnel plot was asymmetry (Fig 7).

### Analysis of Heterogeneity

Heterogeneity has an important influence on the accuracy of the meta-analysis; hence, the reasons for heterogeneity were explored. Generally, variations include several factors such as the cut-off value, study population, measuring methods, study quality (QUADAS) and so on.

Firstly, we explored the threshold effect; we calculated the Spearman correlation coefficient with Moses’ model weighted by inverse variance. These results showed there where no statistically significant difference (Spearman’s correlation coefficient = -0.476, P = 0.233). It meant no threshold effect. Then we used the forest plot of Diagnostic Odds Ratio (Random effects model) to explore the non-threshold effect, the result showed there was non-threshold effect (Cochran-Q = 30.89, P = 0.0001) (Fig 4).

The source of heterogeneity was explored by meta-regression analysis function of Meta-Disc 1.4 software. The process were turn the variations such as cut-off, study quality (QUADAS), and study group from left “Covariates” to the right “Model” to analyze, according the descending P values to remove the covariate and analyze, respectively. The results showed that the main cause of the heterogeneity was the QUADAS of IL-8 test (QUADAS Coeff = 0.699, P = 0.0740 95% CI: 0.20 to 14.32) in Tables 3, 4 and 5.

**Table 3:** Meta-regression analysis of the effects of some covariates on IL-8 in diagnosis of neonatal sepsis.

Covariates	Coefficient	Stand error	RDOR(95%CI)	P value
Cut-off	0.008	0.0041	1.01 (1.00;1.02)	0.1381
QUADAS	0.699	0.2591	2.01 (0.20;14.32)	0.0740
Onset time	1.283	0.7321	3.61 (0.35;37.06)	0.1781

**Table 4:** Meta-regression analysis of the effects of cut-off and QUADAS on IL-8 in diagnosis of neonatal sepsis.

Covariates	Coefficient	Stand error	RDOR(95%CI)	P value
Cut-off	0.011	0.0068	1.01(0.99;1.03)	0.1819
QUADAS	0.854	05068	2.35(0.58;9.60)	0.1671

**Table 5:** Meta-regression analysis of the effects of cut-off and QUADAS on IL-8 in diagnosis of neonatal sepsis.

Covariates	Coefficient	Stand error	RDOR(95%CI)	P value
QUADAS	0.805	0.6066	2.24 (0.47;10.63)	0.2420

The results of sensitivity analysis showed in Table 6.

**Table 6:** Sensitivity analysis of the meta-analysis.

Studies	DOR(95% CI)	Cochrane-Q(p)	AUC	Q*
8 studies	21.64(7.37;63.54)	30.89(0.0001)	0.8908	0.8215
7studies (excluded the cut-off = 0.65 pg/ml)	30.23(9.35;97.69)	26.01(0.0002)	0.8991	0.8303

The results of FPRP analysis showed that all FPRP were less than 0.01 at various priors, which means that the FPRP is low and our finding is noteworthy.

## Discussion

Neonatal sepsis is a life-threatening disorder and an important cause of morbidity and mortality in neonates. Early clinical signs and symptoms of NS are nonspecific, and it is easy to misdiagnose with other common inflammatory diseases such as pneumonia and respiratory distress syndrome. Furthermore laboratory tests are non-specific; and blood culture is suffered form low sensitivity and time-consuming.

Previous studies showed that interleukin 8 (IL-8) may effectively and rapidly diagnose NS. So we conducted the systematic review and meta-analysis to investigate the diagnostic value of the IL-8 in NS. The pooled sensitivity of IL-8 test for the diagnosis of NS was 0.78 (95% CI: 0.72 to 0.83), pooled specificity was 0.84 (95% CI: 0.79 to 0.88), and I^2^ value were 83.7% and 85.1%, respectively. The overall accuracy of IL-8 test for the diagnosis of NS was favorable (AUC = 0.8908, Q* = 0.8215). These results have shown that the IL-8 has moderate accuracy for the diagnosis of NS; therefore, it is a good biomarker for the early diagnosis of NS.

Based on a systematic review of the global literature, Meem et al have classified IL-8 as an early-phase biomarker for detection of NS [11]. CRP is a traditionally used biomarker, which has been applied for clinical purpose [11, 12, 24]. The sensitivity and specificity of CRP were 41% to 96% and 72% to 100%, respectively [11]. In our meta-analysis, the sensitivity and specificity of IL-8 were 35% to 95% and 67% to 100%, respectively. These studies have shown that IL-8 is similar to CRP; hence, IL-8 is a useful biomarker in the diagnosis of NS. In addition, PCT is a more excellent biomarker which has better accuracy than CRP for the diagnosis of NS [11, 25, 26]. In our meta-analysis, the pooled sensitivity (0.78 versus 0.81) of IL-8 was slightly lower than that of the PCT test in NS, and the pooled specificity was higher than PCT (0.84 versus 0.79). The AUC of IL-8 was similar to PCT (0.8908 versus 0.899) [11]. Generally these results have suggested that IL-8 is a useful biomarker for the early diagnosis of NS.

Certainly, our meta-analysis has several limitations. First of all, we considered the existed of heterogeneity. Threshold effect analysis showed there was no threshold effect. The results of DOR showed the existed of non-threshold effect. Meta-regression have proved that the diagnostic accuracy of IL-8 test for predicting NS was not affected by the quality of study (QUADAS), cut-off and time of sepsis onset. We did not made sub-analysis, because the studies group including EONS and LONS, and the proportion of it was different. However the cut-off values of IL-8 widely ranged from 0.65 to 300 pg/ml [12, 24, 27–32]. We thought the difference in the cut-off might due to measure method and the onset time of neonatal sepsis. The table 1 showed the articles differ in proportion of early onset neonatal sepsis (EONS<72 hours after born) and late onset neonatal sepsis (LONS>72 hours after born), and some articles only contain EONE or LONS. The organisms associated with EONS and LONS are different, Group B streptococcus is a gram-positive bacterium which leading to EONS, and 70% of first episode late-onset infections were caused by gram-positive organisms, with coagulase-negative staphylococci accounting for 48% of the infections. Because different of early-onset and late-onset neonatal sepsis pathogens, different degrees of the inflammatory response, and the cut-off different between EONS and LONS [33]. And other unrecorded difference between the articles such as reagents has effect on cut-off which could also lead to heterogeneity. In order to solve the problem, we did sensitivity analysis to explore the stability of cut-off. When we excluded the articles which studied by Kocabas (cut-off = 0.65 pg/ml), the DOR were 21.64 (7.37; 63.54) and 30.23 (9.35; 97.69), respectively; the results showed that the DOR of the meta-analysis was overlapped. And the area under curve 0.8908 (Q* = 0.8215) was similar to 0.8991 (Q* = 0.8303), when excluded the articles studied by Kocabas. These two results showed that the article whose cut-off equal 0.65pg/ml had small effect on the diagnostic accuracy of IL-8 for predicting neonatal sepsis [12, 24, 27–32]. In order to solve this problem we could study homogenous population, but selection bias might significant.

We detected the publication bias of the meta-analysis. As we all know, articles with positive results are more likely to be published, comes the problem of overestimation of the diagnostic accuracy [34]. In order to solve this problem, we searched the databases for further articles and reference lists of primary studies, however there were no additional relevant articles. Publication bias was generally difficult to be avoided during meta-analysis; more studies should be included.

The FPRP analysis shows very low probability of false positive finding in DOR obtained from our meta-analysis. Based on our pre-set criteria of FPRP<0.2, our meta-analysis result is noteworthy.

The results showed that IL-8 was a useful biomarker for detecting neonatal sepsis. Neonatal sepsis was an innate immunological response of systemic inflammation to infection. A singular ideal biomarker has not yet been identified [35]; IL-8 evaluates promptly within 1–3 hours of infection and its half-life is less than 4 hours [9–11]. So IL-8 is one of the most promising biomarker for early diagnosis of neonatal sepsis.

Because the sample size of included studies was relatively small, further studies with big sample size were needed to reduce heterogeneity for IL-8 as a diagnostic biomarker on NS to make meta-analysis more convenient.

## Conclusion

In summary, IL-8 is a helpful biomarker for diagnosis of NS in ill neonates. IL-8 is considered for early diagnosis of NS. Due to insufficient testing data, the experiment results need continuous re-evaluation and clinical validation.

## Supporting Information

S1 Text PRISMA checklist. (DOC)

S2 Text PRISMA 2009 Flow Diagram. (DOC)

## References

[1] Kaistha N, Mehta M, Singla N, Garg R, Chander J. Neonatal septicemia isolates and resistance patterns in a tertiary care hospital of North India. J Infect Dev Ctries. 2009 11 13; 4(1):55–7 . 20130381 10.3855/jidc.625

[2] Blencowe H, Vos T, Lee AC, Philips R, Lozano R, Alvarado MR, et al Estimates of neonatal morbidities and disabilities at regional and global levels for 2010: introduction, methods overview, and relevant findings from the Global Burden of Disease study. Pediatr Res. 2013 12; 74 Suppl 1:4–16. doi: 10.1038/pr.2013.203 24366460; PubMed Central PMCID: PMC3873708. 24366460 10.1038/pr.2013.203 PMC3873708

[3] Remington J, Klein J. Current concepts of infections of the fetus and newborn infant In: JS R, JO K, editors. Infectious Diseases of the Fetus and Newborn Infants. Philadelphia: PA: WB Saunders; 1995pp. 1–19.

[4] Jyothi P, Basavaraj MC, Basavaraj PV. Bacteriological profile of neonatal septicemia and antibiotic susceptibility pattern of the isolates. J Nat Sci Biol Med. 2013 7; 4(2):306–9. 10.4103/0976-9668.116981 24082722 PMC3783770

[5] Radulova P. Neonatal infections. Diagnostic markers of infection. Akush Ginekol (Sofiia). 2010; 49(5):42–51. Review. Bulgarian . 21268402

[6] Clark RH, Bloom BT, Spitzer AR, Gerstmann DR. Reported medication use in the neonatal intensive care unit: data from a large national data set. Pediatrics. 2006 6; 117(6):1979–87 . 16740839 10.1542/peds.2005-1707

[7] Simonsen KA, Anderson-Berry AL, Delair SF, Davies HD. Early-onset neonatal sepsis. Clin Microbiol Rev. 2014 1; 27(1):21–47. 10.1128/CMR.00031-13 Review 24396135 PMC3910904

[8] Mishra UK, Jacobs SE, Doyle LW, Garland SM. Newer approaches to the diagnosis of early onset neonatal sepsis. Arch Dis Child Fetal Neonatal Ed. 2006 5; 91(3):F208–12. Review 16632649 10.1136/adc.2004.064188 PMC2672708

[9] Hotoura E, Giapros V, Kostoula A, Spyrou P, Andronikou S. Pre-inflammatory mediators and lymphocyte subpopulations in preterm neonates with sepsis. Inflammation. 2012 6; 35(3):1094–101. 10.1007/s10753-011-9416-3 . 22160841

[10] Baggiolini M, Walz A, Kunkel SL. Neutrophil-activating peptide-1/interleukin 8, a novel cytokine that activates neutrophils. J Clin Invest. 1989 10; 84(4):1045–9 2677047 10.1172/JCI114265 PMC329758

[11] Meem M, Modak JK, Mortuza R, Morshed M, Islam MS, Saha SK. Biomarkers for diagnosis of neonatal infections: A systematic analysis of their potential as a point-of-care diagnostics. J Glob Health. 2011 12; 1(2):201–9 23198119 PMC3484777

[12] Prashant A, Vishwanath P, Kulkarni P, Sathya Narayana P, Gowdara V, Nataraj SM, et al Comparative assessment of cytokines and other inflammatory markers for the early diagnosis of neonatal sepsis-a case control study. PLoS One. 2013 7 15; 8(7):e68426 10.1371/journal.pone.0068426 PubMed Central PMCID: PMC3711816. 23869218 PMC3711816

[13] Whiting P, Rutjes AW, Reitsma JB, Bossuyt PM, Kleijnen J. The development of QUADAS: a tool for the quality assessment of studies of diagnostic accuracy included in systematic reviews. BMC Med Res Methodol. 2003 11 10; 3:25 14606960 10.1186/1471-2288-3-25 PMC305345

[14] Zamora J, Abraira V, Muriel A, Khan K, Coomarasamy A. Meta-DiSc: a software for meta-analysis of test accuracy data. BMC Med Res Methodol. 2006 7 12; 6:31 16836745 10.1186/1471-2288-6-31 PMC1552081

[15] Devillé WL, Buntinx F, Bouter LM, Montori VM, de Vet HC, van der Windt DA, et al Conducting systematic reviews of diagnostic studies: didactic guidelines. BMC Med Res Methodol. 2002 7 3; 2:9 12097142 10.1186/1471-2288-2-9 PMC117243

[16] Irwig L, Tosteson AN, Gatsonis C, Lau J, Colditz G, Chalmers TC, et al Guidelines for meta-analyses evaluating diagnostic tests. Ann Intern Med. 1994 4 15; 120(8):667–76 . 8135452 10.7326/0003-4819-120-8-199404150-00008

[17] Vamvakas EC. Meta-analyses of studies of the diagnostic accuracy of laboratory tests: a review of the concepts and methods. Arch Pathol Lab Med. 1998 8; 122(8):675–86 . 9701328

[18] Walter SD. Properties of the summary receiver operating characteristic (SROC) curve for diagnostic test data. Stat Med. 2002 5 15; 21(9):1237–56 . 12111876 10.1002/sim.1099

[19] Arends LR, Hamza TH, van Houwelingen JC, Heijenbrok-Kal MH, Hunink MG, Stijnen T. Bivariate random effects meta-analysis of ROC curves. Med Decis Making. 2008 Sep-Oct; 28(5):621–38. 10.1177/0272989X08319957 Epub 2008 Jun 30 . 18591542

[20] Chappell FM, Raab GM, Wardlaw JM. When are summary ROC curves appropriate for diagnostic meta-analyses? Stat Med. 2009 9 20; 28(21):2653–68. 10.1002/sim.3631 . 19591118

[21] Moses LE, Shapiro D, Littenberg B. Combining independent studies of a diagnostic test into a summary ROC curve: data-analytic approaches and some additional considerations. Stat Med. 1993 7 30; 12(14):1293–316 . 8210827 10.1002/sim.4780121403

[22] He J, Xu Y, Qiu LX, Li J, Zhou XY, Sun MH, et al Polymorphisms in ERCC1 and XPF genes and risk of gastric cancer in an eastern Chinese population. PLoS One. 2012; 7(11):e49308 10.1371/journal.pone.0049308 23166636 PMC3499547

[23] Wacholder S, Chanock S, Garcia-Closas M, El Ghormli L, Rothman N. Assessing the probability that a positive report is false: an approach for molecular epidemiology studies. J Natl Cancer Inst. 2004 3 17; 96(6):434–42 . 15026468 10.1093/jnci/djh075 PMC7713993

[24] Boskabadi H, Maamouri G, Afshari JT, Ghayour-Mobarhan M, Shakeri MT. Serum interleukin 8 level as a diagnostic marker in late neonatal sepsis. Iran J Pediatr. 2010 3; 20(1):41–7 23056680 PMC3446011

[25] Vouloumanou EK, Plessa E, Karageorgopoulos DE, Mantadakis E, Falagas ME. Serum procalcitonin as a diagnostic marker for neonatal sepsis: a systematic review and meta-analysis. Intensive Care Med. 2011 5; 37(5):747–62. 10.1007/s00134-011-2174-8 Epub 2011 Mar 5 . 21380522

[26] Yu Z, Liu J, Sun Q, Qiu Y, Han S, Guo X. The accuracy of the procalcitonin test for the diagnosis of neonatal sepsis: a meta-analysis. Scand J Infect Dis. 2010 10; 42(10):723–33. 10.3109/00365548.2010.489906 . 20840003

[27] Kocabaş E, Sarikçioğlu A, Aksaray N, Seydaoğlu G, Seyhun Y, Yaman A. Role of procalcitonin, C-reactive protein, interleukin-6, interleukin-8 and tumor necrosis factor-alpha in the diagnosis of neonatal sepsis. Turk J Pediatr. 2007 Jan-Mar; 49(1):7–20 . 17479639

[28] Laborada G, Rego M, Jain A, Guliano M, Stavola J, Ballabh P, et al Diagnostic value of cytokines and C-reactive protein in the first 24 hours of neonatal sepsis. Am J Perinatol. 2003 11; 20(8):491–501 . 14703598 10.1055/s-2003-45382

[29] Santana Reyes C, García-Muñoz F, Reyes D, González G, Dominguez C, Domenech E. Role of cytokines (interleukin-1beta, 6, 8, tumour necrosis factor-alpha, and soluble receptor of interleukin-2) and C-reactive protein in the diagnosis of neonatal sepsis. Acta Paediatr. 2003; 92(2):221–7 . 12710650 10.1111/j.1651-2227.2003.tb00530.x

[30] Martin H, Olander B, Norman M. Reactive hyperemia and interleukin 6, interleukin 8, and tumor necrosis factor-alpha in the diagnosis of early-onset neonatal sepsis. Pediatrics. 2001 10; 108(4):E61 . 11581469 10.1542/peds.108.4.e61

[31] Nupponen I, Andersson S, Järvenpää AL, Kautiainen H, Repo H. Neutrophil CD11b expression and circulating interleukin-8 as diagnostic markers for early-onset neonatal sepsis. Pediatrics. 2001 7; 108(1):E12 . 11433091 10.1542/peds.108.1.e12

[32] Berner R, Niemeyer CM, Leititis JU, Funke A, Schwab C, Rau U, et al Plasma levels and gene expression of granulocyte colony-stimulating factor, tumor necrosis factor-alpha, interleukin (IL)-1beta, IL-6, IL-8, and soluble intercellular adhesion molecule-1 in neonatal early onset sepsis. Pediatr Res. 1998 10; 44(4):469–77 . 9773833 10.1203/00006450-199810000-00002

[33] Shah BA, Padbury JF. Neonatal sepsis: an old problem with new insights. Virulence. 2014 1 1; 5(1):170–8. 10.4161/viru.26906 24185532 PMC3916371

[34] Thornton A, Lee P. Publication bias in meta-analysis: its causes and consequences. J Clin Epidemiol. 2000 2; 53(2):207–16 . 10729693 10.1016/s0895-4356(99)00161-4

[35] Chan T, Gu F. Early diagnosis of sepsis using serum biomarkers. Expert Rev Mol Diagn. 2011 6; 11(5):487–96. 10.1586/ERM.11.26 . 21707457

