# Supplementary material for: Interleukin-8 for Diagnosis of Neonatal Sepsis: A Meta-Analysis
Source: PLoS One. 2015 May 21;10(5):e0127170. doi: 10.1371/journal.pone.0127170 (PMC4440704; doi:10.1371/journal.pone.0127170)
Supplement: S2 Text — (DOC) [file pone.0127170.s002.doc]

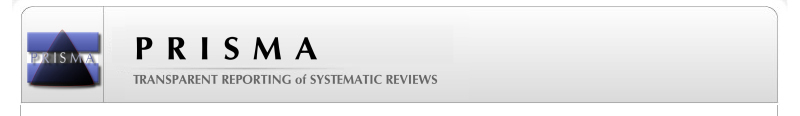
**PRISMA 2009 Flow Diagram**

**Screening**

**Included**

**Eligibility**

**Identification**

Records identified through database searching
(n = 802 )

Records after duplicates removed
(n = 802 )

Records screened
(n = 47 )

Records excluded
(n = 755 )

Full-text articles assessed for eligibility
(n = 22 )

Full-text articles excluded, with reasons
(n = 25 )

Studies included in qualitative synthesis
(n = 8 )

Studies included in quantitative synthesis (meta-analysis)
(n = 8)
